# Transparent Superhydrophobic and Self-Cleaning Coating

**DOI:** 10.3390/polym16131876

**Published:** 2024-07-01

**Authors:** Binbin Zhang, Xiaochen Xue, Lixia Zhao, Baorong Hou

**Affiliations:** 1 Key Laboratory of Advanced Marine Materials, Key Laboratory of Marine Environmental Corrosion and Bio-Fouling, Institute of Oceanology, Chinese Academy of Sciences, Qingdao 266071, China; 2 University of Chinese Academy of Sciences, Beijing 100049, China

**Keywords:** superhydrophobic, self-cleaning, transparent, water repellency

## Abstract

Surface roughness and low surface energy are key elements for the artificial preparation of biomimetic superhydrophobic materials. However, the presence of micro-/nanostructures and the corresponding increase in roughness can increase light scattering, thereby reducing the surface transparency. Therefore, designing and constructing superhydrophobic surfaces that combine superhydrophobicity with high transparency has been a continuous research focus for researchers and engineers. In this study, a transparent superhydrophobic coating was constructed on glass substrates using hydrophobic fumed silica (HF-SiO_2_) and waterborne polyurethane (WPU) as raw materials, combined with a simple spray-coating technique, resulting in a water contact angle (WCA) of 158.7 ± 1.5° and a sliding angle (SA) of 6.2 ± 1.8°. Characterization tests including SEM, EDS, LSCM, FTIR, and XPS revealed the presence of micron-scale protrusions and a nano-scale porous network composite structure on the surface. The presence of HF-SiO_2_ not only provided a certain roughness but also effectively reduced surface energy. More importantly, the coating exhibited excellent water-repellent properties, extremely low interfacial adhesion, self-cleaning ability, and high transparency, with the light transmittance of the coated glass substrate reaching 96.1% of that of the bare glass substrate. The series of functional characteristics demonstrated by the transparent superhydrophobic HF-SiO_2_@WPU coating designed and constructed in this study will play an important role in various applications such as underwater observation windows, building glass facades, automotive glass, and goggles.

## 1. Introduction

For numerous optical devices and high-precision instruments, the issue of surface adhesion of airborne microparticles, dust, or tiny droplets, along with the adhesion of microorganisms underwater, commonly arises during operation, impacting the devices’ normal functionality [1,2]. The cleaning and maintenance procedures are typically time-consuming and labor-intensive and can easily harm the surface optical properties of the devices. In nature, the surface of lotus leaves is not easily contaminated by pollutants. Even in the presence of solid or liquid pollutants, they can remain clean and tidy under the action of water droplets or water flow, a phenomenon known as the self-cleaning lotus effect. The self-cleaning effect is mainly attributed to the multi-level micro-/nanostructure and low-surface-energy wax layer on the surface of lotus leaves, giving them superhydrophobic properties. Typically, researchers define surfaces with a WCA greater than 150º and an SA less than 10° as superhydrophobic materials [3,4]. The distinctive non-wetting interface and low surface energy properties of superhydrophobic materials have garnered significant across various application domains, notably in anti-bacterial [5,6], anti-corrosion [7,8,9], and anti-icing measures [10,11]; oil–water separation [12,13]; self-cleaning [14,15]; flame retardancy [16,17]; wearable sensors [18,19]; medical devices [20,21]; energy harvesting [22,23]; and so on.

The development of superhydrophobic coatings with high transmittance can prevent pollutant attachment; repel rainwater and fog; and preserve aesthetic appeal and transparency, crucial in domains such as solar panels, safety goggles, architectural glass, automotive glass, underwater observation windows, building facades, and electronic devices [24,25]. For solar panels, establishing a highly transparent superhydrophobic coating on the surface can significantly reduce the labor and costs associated with manual cleaning while maintaining the energy collection efficiency and lowering the cleaning maintenance frequency. In the case of underwater observation windows, a transparent superhydrophobic coating can minimize microbial attachment underwater while preserving observation capabilities. For automobile glass, a surface with high transparency and superhydrophobic properties can always remain clean without affecting visibility. These instances underscore the substantial potential and necessity for the advancement of highly transparent superhydrophobic materials in diverse fields.

Over the past two decades, researchers have made significant progress in the study of artificially prepared superhydrophobic materials. They have proposed various methods for preparing superhydrophobic materials by enhancing roughness and reducing surface energy, including chemical etching, anodization, laser treatment, electrodeposition, spin coating, electrospinning, spray coating, and more. It is widely recognized that the surface’s micro/nano multi-scale rough structure and low surface energy are fundamental components for achieving superhydrophobic materials [26,27,28]. Nevertheless, heightened surface roughness can amplify light scattering, thereby influencing the light-transmission performance of superhydrophobic coatings [29,30,31]. Consequently, the challenge of designing transparent or semi-transparent superhydrophobic surfaces on a glass substrate that exhibit both superhydrophobicity and high light transmission has been a central focus in the realm of superhydrophobic functional protective materials. To date, a variety of top-down and bottom-up methods have been explored to create superhydrophobic surfaces with high transparency. For instance, Ebert et al. [32] reported a transparent superhydrophobic surface produced through O_2_/CF_4_ etching followed by an additional treatment with octafluorocyclobutane (C_4_F_8_) plasma or vapor deposition of perfluorooctyltrichlorosilane (PFOTCS). Martin et al. [33] fabricated a micropatterned superhydrophobic PDMS sample with high transmission using a four-step soft-lithography technique. Rao et al. [34] prepared an optically transparent superhydrophobic silica film using the sol–gel process with the dip-coating technique. Mates et al. [35] spray-coated an entirely water-based and non-fluorinated transparent superhydrophobic coating from hydrophilic titanium dioxide (TiO_2_) nanoparticles and polyolefin copolymers, without additional surfactants or charge stabilization. Li et al. [36] prepared a transparent superhydrophobic coating based on SiO_2_ nanoparticles combined with NH_2_-terminated silicone or SN_2_-modified polyurethane by alternately spin-coating them onto glass slides. Comparing the techniques documented in the current literature for crafting highly transparent superhydrophobic materials indicates that most preparation methods suffer from complexity, time intensiveness, high costs, and ecological impact. Thus, there exists a pressing need to identify a straightforward, effective, cost-efficient method with broad substrate applicability to acquire transparent or semi-transparent superhydrophobic materials.

Hydrophobic fumed silica (HF-SiO_2_) is a white, fluffy, amorphous powder with several advantages, such as being lightweight and porous, possessing a large specific surface area, exhibiting good chemical stability, and being cost-effective. These characteristics make it an ideal raw material in the fields of gas separation [37,38], functional coatings [39,40], adhesives [41,42], and more. Additionally, the surface of HF-SiO_2_ nanoparticles is coated with low-surface-energy molecules. This feature not only provides a certain roughness when used as a filler in coating material preparation but also effectively reduces surface energy. As a result, HF-SiO_2_ has garnered significant attention and application in the development of superhydrophobic materials [43,44,45,46]. However, research on HF-SiO_2_ in the design and application of transparent or semi-transparent superhydrophobic materials has been relatively limited.

In this study, HF-SiO_2_ and waterborne polyurethane (WPU) were employed as raw materials to design and fabricate a superhydrophobic coating with high transmittance on a glass substrate using a simple and substrate-independent spray-coating technique. The surface micromorphology, elemental composition, non-wettability behavior, light transmittance, and self-cleaning properties of the coating were thoroughly investigated. The results indicated that the coating demonstrated excellent superhydrophobic properties and high light transmittance, successfully overcoming the challenge of these two characteristics typically being mutually exclusive. Additionally, the dynamic droplet bouncing behavior and self-cleaning properties exhibited by the coating offer functional assurance for its extensive application on various optical devices and surfaces.

## 2. Experimental Section

### 2.1. Materials and Reagents

Microscope slides (CAT.NO.7101, SAIL BRAND, Huddersfield, UK) were used as glass substrates with a size of 76.2 mm × 25.4 mm × 1.2 mm. Waterborne polyurethane (WPU-2830) was obtained from Dongguan Haosheng New Materials Co., Ltd, Dongguan, China. Hydrophobic fumed nano-silica (HF-SiO_2_, R972, 16 nm, ≥99.8%, CAS number: 68611-44-9) was purchased from AEROSIL, Essen, Germany. Absolute ethanol (CH_3_CH_2_OH, ≥ 99.7%, CAS number: 64-17-5), methylene blue trihydrate (C_16_H_18_CIN_3_S•3H_2_O, 99.5%, CAS number: 7220-79-3), potassium chromate (K_2_CrO_4_, 99.0%, CAS number: 7789-00-6), copper (II) chloride dihydrate (CuCl_2_·2H_2_O, 99.0%, CAS number: 10125-13-0), and silicon carbide (SiC, 97.5%, CAS number: 409-21-2) were provided by Sinopharm Chemical Reagent Co., Ltd, Beijing, China. All reagents used in the experiment were of analytical grade without further purification.

### 2.2. Fabrication of Transparent Superhydrophobic Coating

**Figure 1:**
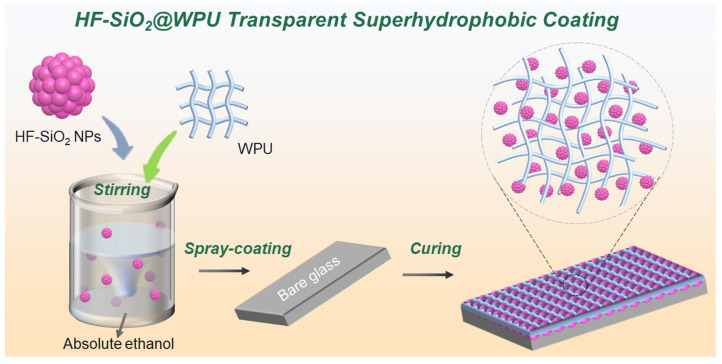
Schematic illustration of the preparation process of HF-SiO_2_@WPU transparent superhydrophobic coating.

The bare glass substrate was cleaned with absolute ethanol before usage. HF-SiO_2_ nanoparticles with a total mass of 0.15 g were dispersed in 5 mL absolute ethanol under constant stirring for 30 min at room temperature (298 K) to obtain a uniform suspension, designated suspension A. A mass of 0.15 g WPU was dissolved in 5 mL absolute ethanol and magnetically stirred for 30 min at room temperature (298 K) to obtain suspension B. Then, suspension A and suspension B were mixed under magnetic stirring for 30 min at room temperature (298 K) to obtain the HF-SiO_2_@WPU suspension. The uniform HF-SiO_2_@WPU suspension was sprayed onto the cleaned glass substrates under 0.18 MPa air pressure. The distance between the glass substrates and the spray gun was ~15 cm. The spray-coated substrate was cured at 120 °C for 1 h to produce the testing sample. A schematic illustration of the fabrication of the HF-SiO_2_@WPU transparent superhydrophobic coating is shown in Figure 1.

### 2.3. Characterizations

The surface morphology and roughness of the coatings were investigated using a scanning electronic microscope (FE-SEM, FEI Nova NanoSEM 450, Waltham, MA, USA) and a laser scanning confocal microscope (LSCM, OLS5000, Olympus, Tokyo, Japan). The chemical compositions of the samples were analyzed using energy-dispersive spectrometry (EDS), X-ray photoelectron spectroscopy (XPS, Thermo Scientific Escalab 250Xi, Waltham, USA), and Fourier transform infrared spectrometry (FT-IR, Thermo Scientific NICOLET iS10, Waltham, MA, USA). A UV–Vis diffuse reflectance spectrophotometer (Hitachi U-3900H, Tokyo, Japan) was used to measure the transmittance of the HF-SiO_2_@WPU superhydrophobic coating. A contact angle measuring instrument (Dataphysics OCA 25, Stuttgart, Germany) was used to measure water contact angles (WCAs) and sliding angles (SAs) at different locations on the samples. In the WCA and SA tests, five different test points were selected on different sample surfaces, and the average values were calculated. The bouncing behavior of water droplets on the bare glass and the HF-SiO_2_@WPU transparent superhydrophobic coatings was recorded by using a high-speed camera (Photron, FASTCAM Mini UX100, Tokyo, Japan). The liquid volume in the water droplet impact test was 10 μL with a drop height of 1 cm.

## 3. Results and Discussion

### 3.1. Surface Morphologies and Chemical Compositions

**Figure 2:**
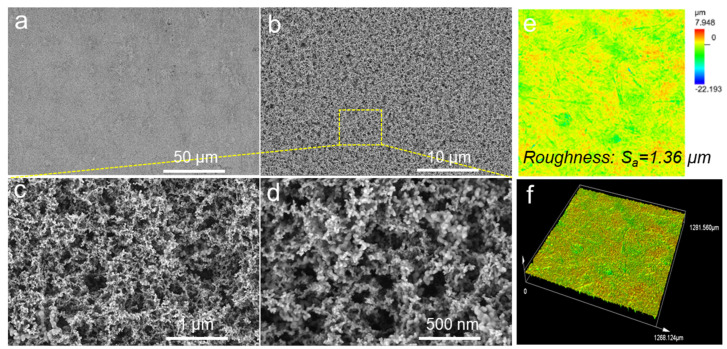
SEM images of the (**a**) bare glass and (**b**–**d**) prepared HF-SiO_2_@WPU superhydrophobic coating under different magnifications; (**e**,**f**) 2D and 3D LSCM images of the fabricated HF-SiO_2_@WPU superhydrophobic coating.

Figure 2a shows an SEM image of the bare glass substrate; the surface is very smooth and flat. Figure 2b–d show SEM images of the prepared HF-SiO_2_@WPU superhydrophobic coating. Many micron-level protrusions can be observed on the surface at low magnification, and a nanoporous network structure can be observed at high magnification. Additionally, a laser scanning confocal microscope was employed to observe the surface roughness of the prepared HF-SiO_2_@WPU superhydrophobic coating. The 2D and 3D images are shown in Figure 2e and Figure 2f, respectively. The surface roughness average (S_a_) of the constructed HF-SiO_2_@WPU superhydrophobic coating was 1.36 μm. The appearance of these micron protrusions and nanoporous structures provided a certain rough structure to the surface, and the gaps between these rough structures provided storage space for trapping a large amount of air, which is one of the core elements that enable the surface to exhibit superhydrophobic properties with Cassie–Baxter interface contact. Additionally, the nano-scale porous structures also benefit the light transmission because they are smaller than the quarter-wavelength of visible light.

**Figure 3:**
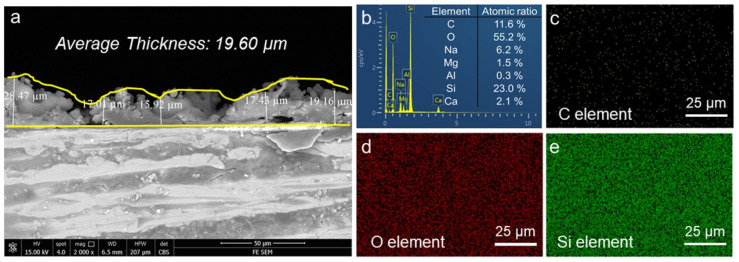
(**a**) Cross-section SEM image, (**b**) EDS spectra, and (**c**–**e**) EDS elemental mappings of the HF-SiO_2_@WPU superhydrophobic coating.

To obtain the thickness value, a cross-section SEM image was recorded, as shown in Figure 3a. Five different positions were marked to calculate the average thickness of the prepared HF-SiO_2_@WPU superhydrophobic coating. The average thickness of the coating was 19.60 μm. Figure 3b show the EDS spectra of HF-SiO_2_@WPU superhydrophobic coatings. The surface of HF-SiO_2_@WPU superhydrophobic coating mainly contains three elements, namely, C, O, and Si, with atomic ratios of 11.6%, 55.2%, and 23.0%, respectively. In addition, the occurrence of Na, Mg, Ca, and Al elements in EDS spectra mainly originates from the glass matrix, as the prepared HF-SiO_2_@WPU superhydrophobic coating is very thin and contains numerous micro-/nanostructural gaps. According to the EDS elemental mappings shown in Figure 3c–e, it can be observed that the elements were uniformly distributed on the surface, indicating a uniform HF-SiO_2_@WPU superhydrophobic coating. In general, improving the uniformity of the coating covering the glass helps to achieve high optical transmission.

**Figure 4:**
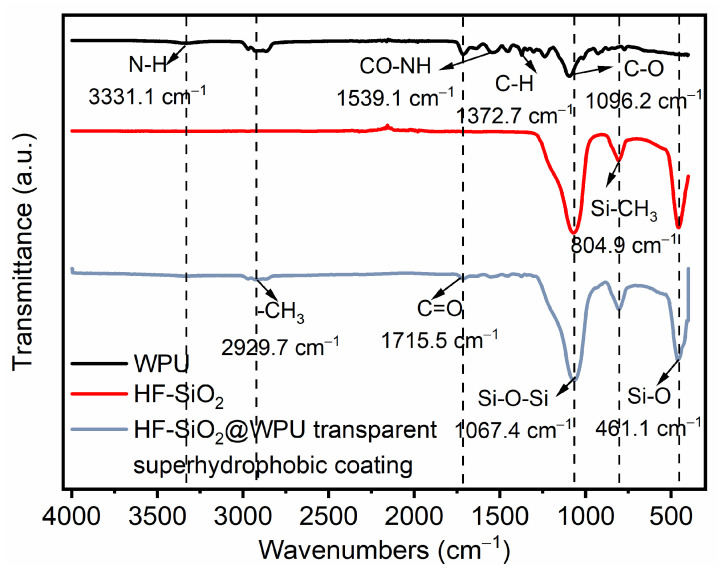
FTIR spectra of WPU, HF-SiO_2_, and HF-SiO_2_@WPU superhydrophobic coating.

FTIR spectra were employed to investigate the chemical groups of WPU, HF-SiO_2_, and HF-SiO_2_@WPU superhydrophobic coating, as shown in Figure 4. The absorption peaks at 2929.7 cm^−1^ and 1715.5 cm^−1^ can be attributed to the stretching vibrations of -CH_3_ and C=O groups. In addition, the absorption peak around 1067.4 cm^−1^ could be assigned to the vibration peaks of Si-O-Si bonds. The peak appearing at 804.9 cm^−1^ was attributed to the rocking of CH_3_ in Si-CH_3_, and the peak at 461.1 cm^−1^ corresponded to the symmetric vibration peak of Si-O bond [47,48,49]. All characteristic peaks in the FTIR spectra indicated the existence of WPU and HF-SiO_2_ nanoparticles, which were conductive to improving the roughness and superhydrophobicity of coatings.

**Figure 5:**
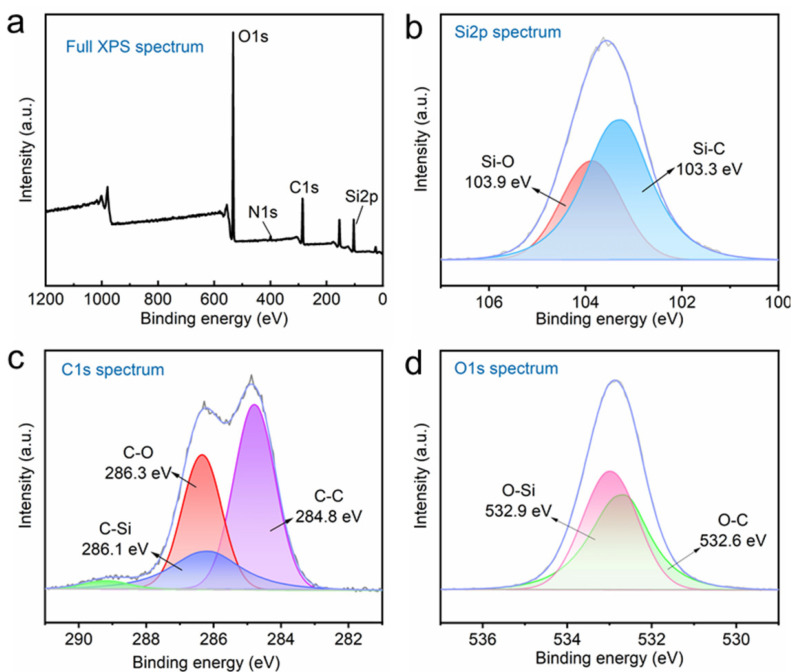
(**a**) Full XPS spectra and high-resolution (**b**) Si2p, (**c**) C1s, and (**d**) O1s spectra of the fabricated HF-SiO_2_@WPU superhydrophobic coating.

To further confirm the elemental composition of the HF-SiO_2_@WPU superhydrophobic coating, X-ray photoelectron spectroscopy (XPS) was used to conform its surface elements. The full XPS spectrum of the HF-SiO_2_@WPU superhydrophobic coating was shown in Figure 5a. The HF-SiO_2_@WPU superhydrophobic coating has four main peaks, including O1s (532.5 eV), N1s (399.3 eV), C1s (284.8 eV), and Si2p (103.2 eV), respectively. Figure 5b–d show the high-resolution Si2p, C1s, and O1s spectra of the HF-SiO_2_@WPU superhydrophobic coating. The C1s spectrum has three peaks with binding energies of 284.8 eV, 286.1 eV, and 286.3 eV, corresponding to C-C, C-Si, and C-O, respectively. For the Si2p spectrum, two peaks at 103.9 eV and 103.3 eV can be assigned to Si-O and Si-C. The O1s spectrum can be fitted to O-Si and O-C peaks, located at 532.9 eV and 532.6 eV, respectively.

### 3.2. Surface Wettability

**Figure 6:**
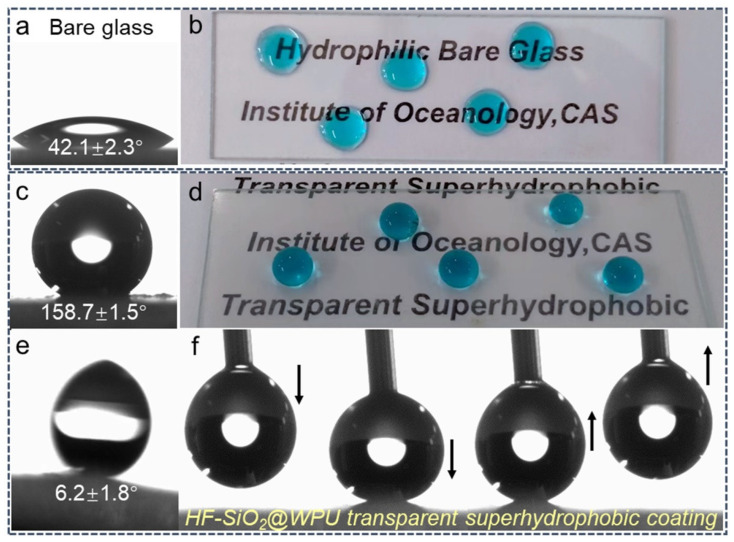
(**a**) WCA image and (**b**) optical images of water droplets on bare glass; (**c**) WCA image and (**d**) optical images of water droplets on the HF-SiO_2_@WPU superhydrophobic coating; (**e**) SA image and (**f**) dynamic water droplet contact behavior on HF-SiO_2_@WPU superhydrophobic surfaces.

Figure 6a shows a contact angle diagram of the bare glass substrate, with a WCA of 42.1 ± 2.3°. Figure 6b shows an optical photograph of dyed water droplets on the surface of a bare microscope slide. The droplets exhibited a Wenzel wetting state on the surface, indicating that the untreated glass substrate is intrinsically hydrophilic. Figure 6c,d show the CA and images of water droplet placed on the HF-SiO_2_@WPU superhydrophobic coating, with a WCA of 158.7 ± 1.5°. On the coated glass surface, the dyed water droplets displayed typical spherical contours, demonstrating a unique Cassie–Baxter non-wetting state, ultra-low liquid–solid contact area, and excellent superhydrophobic properties. Additionally, it could be observed that the coated HF-SiO_2_@WPU superhydrophobic surface presented high transparency. The letters under the coated glass substrate could be easily seen. Figure 6e,f presents the SA and dynamic contact behavior of a water droplet. The SA of the prepared HF-SiO_2_@WPU transparent superhydrophobic coating was 6.2 ± 1.8°. In the dynamic contact testing between the water droplet and the coating surface, we fixed the substrate stage and gradually approached it with a water droplet from a microsyringe. As the droplet contacted the surface, compressed against it, and detached from it, it was observed that the droplet could easily detach from the prepared surface covered with the HF-SiO_2_@WPU transparent superhydrophobic coating without leaving any residual liquid. This indicates that the prepared transparent superhydrophobic coating has extremely low surface energy and reduced interfacial liquid adhesion force, mainly due to the introduction of micro-/nanostructures and low-surface-energy characteristics brought by the HF-SiO_2_ particles.

**Figure 7:**
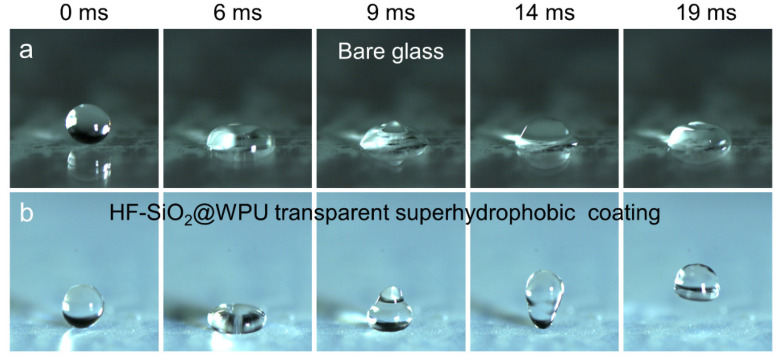
Bouncing behavior and images of water droplets on the (**a**) bare glass and (**b**) HF-SiO_2_@WPU transparent superhydrophobic coating. The volume of the water droplet and the releasing height are 10 μL and 1 cm, respectively.

Figure 7a,b depict images captured by a high-speed camera studying the impact of water droplets on both the bare glass and the glass substrate covered with the coating. For the bare glass substrate, the water droplet exhibited pinning and adhesion upon contact with the surface, displaying a hydrophilic state. In contrast, when the water droplet contacted the surface of the HF-SiO_2_@WPU superhydrophobic coating, it underwent compression, deformation, and bouncing due to the interaction. The solid–liquid contact time was only 14 ms, and the extremely short solid–liquid contact area further demonstrated the excellent superhydrophobic properties of the surface, allowing the water droplet to quickly bounce off the surface after brief contact, leaving no liquid residue. This outstanding superhydrophobic property is mainly attributed to the micro/nano binary hierarchical structure of the HF-SiO_2_@WPU coating surface and the low surface energy of HF-SiO_2_.

### 3.3. Optical Transparency and Self-Cleaning Ability

**Figure 8:**
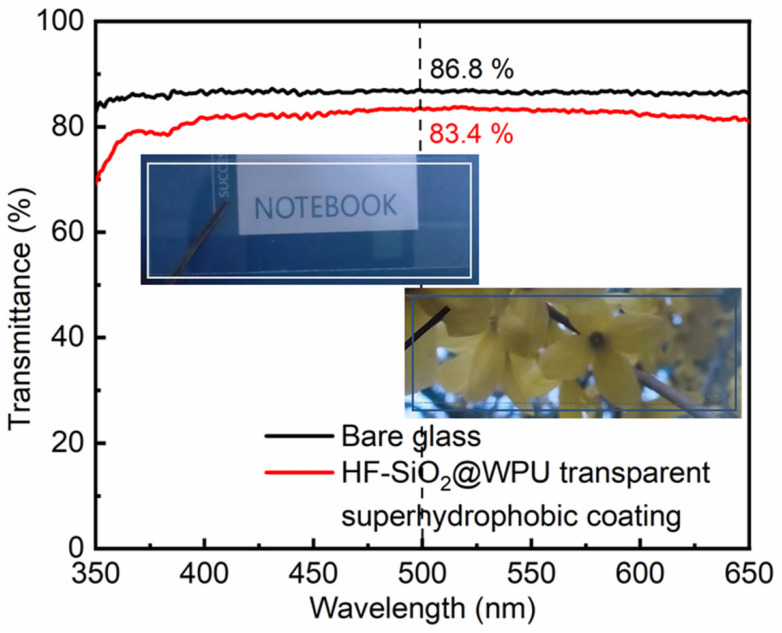
Comparison of the optical transparency properties of the bare glass and fabricated HF-SiO_2_@WPU superhydrophobic coating.

Figure 8 shows the comparison of the light transmittance data between the bare glass substrate and the prepared HF-SiO_2_@WPU superhydrophobic coating. It can be seen from the figure that the light transmittance of the bare glass substrate was 86.8% (wavelength: 500 nm), while the light transmittance of the HF-SiO_2_@WPU superhydrophobic coating was 83.4% (wavelength: 500 nm). By calculating the ratio of the transmittance of the coating to the transmittance of the bare glass substrate, it can be concluded that the light transmittance of the prepared HF-SiO_2_@WPU superhydrophobic coating can reach 96.1% of that of the bare glass substrate, fully confirming the extremely high light transmittance exhibited by the prepared coating. From the inset of Figure 8, it can also be observed that when observing the notebook and yellow flowers through the HF-SiO_2_@WPU superhydrophobic coating-treated microscope slide, the text on the notebook and the structural characteristics of the flowers could be clearly seen. This further confirms that the prepared transparent superhydrophobic coating exhibits extremely high light transmittance. This high light transmittance is mainly due to the very uniform and small-scale nanoporous network structure on the surface, as well as the relatively low thickness of the coating, which is conducive to light transmission. Reducing the thickness of superhydrophobic coatings, decreasing the scale of micro-/nanostructures, and other methods are effective ways to enhance the light transmittance of superhydrophobic coatings. Continuously improving the transparency of the surface while maintaining its superhydrophobic properties will also give this class of materials greater potential for applications in various fields. Additionally, research on the mechanical stability of transparent superhydrophobic coatings is currently receiving widespread attention, as this is also an important factor for transitioning these materials from laboratory design to practical applications.

**Figure 9:**
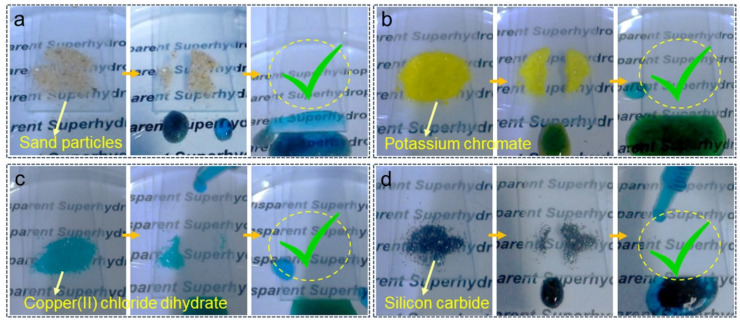
Self-cleaning ability of the fabricated HF-SiO_2_@WPU transparent superhydrophobic coating using (**a**) sand particles, (**b**) potassium chromate, (**c**) copper (II) chloride dihydrate, and (**d**) silicon carbide as solid contaminants.

A self-cleaning test of the HF-SiO_2_@WPU transparent superhydrophobic coatings was carried out by using sand particles, potassium chromate, Copper(II) chloride dihydrate, and silicon carbide as solid contaminants, as shown in Figure 9a–d. Four kinds of solid contaminants were spread abundantly on the coated glass surface. Then, water droplets stained with methylene blue were dropped on the surface area with contaminants. It can be observed that as water droplets were continuously added onto the pollutants, the surface contaminants were removed as the water droplets rolled, replicating the self-cleaning phenomenon observed on lotus leaves. Once the pollutants were completely removed from the surface, it returned to its initial clean state without any liquid or solid residue left. This further demonstrates the excellent self-cleaning functionality conferred by the superhydrophobic nature of the surface, which will be beneficial for its applications in various fields requiring high light transmittance and self-cleaning surfaces, such as architectural glass facades and solar panels.

## 4. Conclusions

In summary, based on the low light transmittance of superhydrophobic materials, achieving the dual functionality of “transparent + superhydrophobic” is challenging. In this research work, we utilized a simple and versatile spray-coating technique to design and prepare a highly transparent superhydrophobic HF-SiO_2_@WPU coating on a microscope slide. The materials used, including HF-SiO_2_ and WPU, are relatively low-cost and environmentally friendly reagents, which will facilitate controlling the material preparation costs and environmental impact. The resulting HF-SiO_2_@WPU transparent superhydrophobic coating shows a hierarchical rough structure, specifically with a nanoporous network structure. These nanostructures not only provide surface roughness but also avoid a sharp increase in roughness, which is beneficial for light transmission through the structure and pores. Various characterization techniques were employed to analyze the morphology and elements on the coating surface, confirming the presence of HF-SiO_2_ and its crucial role in achieving superhydrophobic properties. Transparency tests and comparisons revealed that the prepared HF-SiO_2_@WPU superhydrophobic coating can achieve a light transmittance of 96.1% on bare glass, showcasing exceptionally high light transmission performance. This represents a significant advancement over previously reported transparent and semi-transparent superhydrophobic coatings. Moreover, the coating surface demonstrates outstanding water repellency and self-cleaning properties, as water droplets can bounce off the surface rapidly. High-speed camera tests indicated a solid–liquid contact area of only 14 ms. The superb light transmittance, superhydrophobic characteristics, and self-cleaning capabilities of this coating render it highly promising for a wide array of applications on the surfaces of various optical devices and equipment.

## Data Availability

All data generated or analyzed during this study are included in this published article.
